# Association between self-rated health and the risk of hip fracture and mortality in a cohort of older women during a 10-year follow-up

**DOI:** 10.1371/journal.pone.0247924

**Published:** 2021-03-05

**Authors:** Elin Uzunel, Hans Lundin, Per Wändell, Helena Salminen

**Affiliations:** 1 Department of Neurobiology, Care Sciences and Society, Division of Family Medicine and Primary Care, Karolinska Institutet, Huddinge, Sweden; 2 Academic Primary Health Care Centre, Stockholm, Sweden; Medical College of Wisconsin, UNITED STATES

## Abstract

Fragility fracture of the hip is associated with reduced functional status and mortality. Poor self-rated health (SRH) might be such an indicator. Our aim was to study if SRH was associated with hip fractures and all-cause mortality within the next 10 years in community-dwelling older women. A population-based sample of 350 women aged between 69 and 79 years (median 72.4) assessed their SRH by answering the question “How would you rate your health right now” by putting a mark on a visual-analogue scale (0–100 mm). Information on hip fracture and mortality over the next 10 years was retrieved from health care registers. The association between SRH and hip fracture and all-cause mortality was tested with a Cox proportional hazards regression model. SRH was divided into low, intermediate, and high (reference) assessed SRH. During the study, 40 hip fractures and 72 deaths occurred. The median value of SRH was 62 mm (IQR 50–81 mm). The age-adjusted hazard ratio (HR) for hip fracture was significantly higher in the group with low and intermediate SRH; HR: 3.17 (95% CI 1.25–8.01), and HR: 2.75 (95% CI 1.08–7.04), compared with high SRH. Adding bone mineral density (at the femoral neck) gave even greater risk. We did not find the hypothesized association between SRH and mortality. In our study, SRH indicated a higher risk of future hip fracture in older women. SRH might be a marker that could add information about the risk of hip fracture independently of bone mineral density.

## Introduction

The life-time risk over the age of 50 in Sweden to suffer a hip fracture is 22.9% in women and 10.7% in men [1]. Hip fractures are associated with an increase in mortality, impaired function, and great suffering [2–5] and thus affect quality of life (QoL) negatively [6–8]. The aetiology of osteoporosis is multifactorial and complex, and thus different combinations of risk factors determine the individual risk of fracture in each person [9–13].

QoL is a subjective measure and might be explained as a person´s evaluation of their state of well-being in a general way. It is considered to add complementary information that medical and epidemiological data might not detect [8, 14–16]. Health-related quality of life (HRQoL) is a subset of QoL and might at its simplest be defined as QoL in relation to health status [14–18]. Self-rated health (SRH) is a variant of HRQoL. SRH is a robust health measure and a predictor of mortality and morbidity that can be used as a public health indicator between countries. It is a single-item measure where the person is asked to assess his or her general health, and it is considered to provide summative information about the various domains of health. Various ways of measuring SRH have been described and compared in the literature [19–22]. In this study we used a single question: “How would you rate your health right now?” and the assessment was done by marking along a visual-analogue scale (VAS) ranging from “worst imaginable” to “best imaginable” (0–100 mm). Health-evaluation by VAS is also a well-established but perhaps less frequently used part of a standardized instrument for measuring generic health status (EQ5D) called EQ-VAS [23, 24].

The association between SRH and mortality has been described [25, 26]. That SRH/QoL decreases after a hip fracture may not be surprising. Studies exploring the trajectory of SRH/QoL before hip fracture are not as conclusive [8, 17]. Interestingly a relationship between poor QoL and low bone density (with or without fractures) has also been described [27–30]. It has also been discussed if hip fracture is the event that triggers the decline in SRH or if the decline started years before and the hip fracture rather is a consequence [5, 31].

We hypothesized that SRH might predict future hip fractures and mortality in our pre-existing cohort of elderly and community-dwelling women. This hypothesis is based on the suggestion that there are associations between QoL and bone density/osteoporosis even prior to hip fractures. The aim of the study was to explore how SRH is associated to a) hip fracture or b) death by any cause, independently of each other, during the next ten years. This would also add new knowledge about SRH prior to hip fracture.

## Materials and methods

### Study design

This was a longitudinal cohort study with about 10 years of follow up. The participants were part of a cohort originally gathered as part of the Primary Health Care and Osteoporosis (PRIMOS) project, a series of studies on elderly and community-dwelling women concerning different aspects of osteoporosis. Ethical approval was obtained from the Regional Ethical Review Board in Stockholm (Dnr 145/98, 2007/188-31/3 and 2011/1743-32) and the Radiation Protection Committee at Karolinska University Hospital. All participants gave their written informed consent at baseline. The first woman was recruited in March 1999 and the last in February 2001 and SRH were assessed at enrolment [32–37]. The main outcomes in the present study were hip fracture and overall mortality. The end of the present study was set to December 2009. Data regarding hip fractures and deaths were retrieved retrospectively from the Swedish National Board of Health and Welfare´s National inpatient and outpatient registers and Cause of Death register. Due to the comprehensiveness of those registers, there was no loss of follow-up [38, 39]. Participants were contributing to the time at risk of hip fracture until they suffered a hip fractur, died or reached the end date of the study. Regarding all-cause mortality the participants that suffered a hip fracture were still considered at risk until they died of any cause or reached the end of the study. Some of the data are re-analyzed to answer our research questions.

### Participants

**Fig 1:**
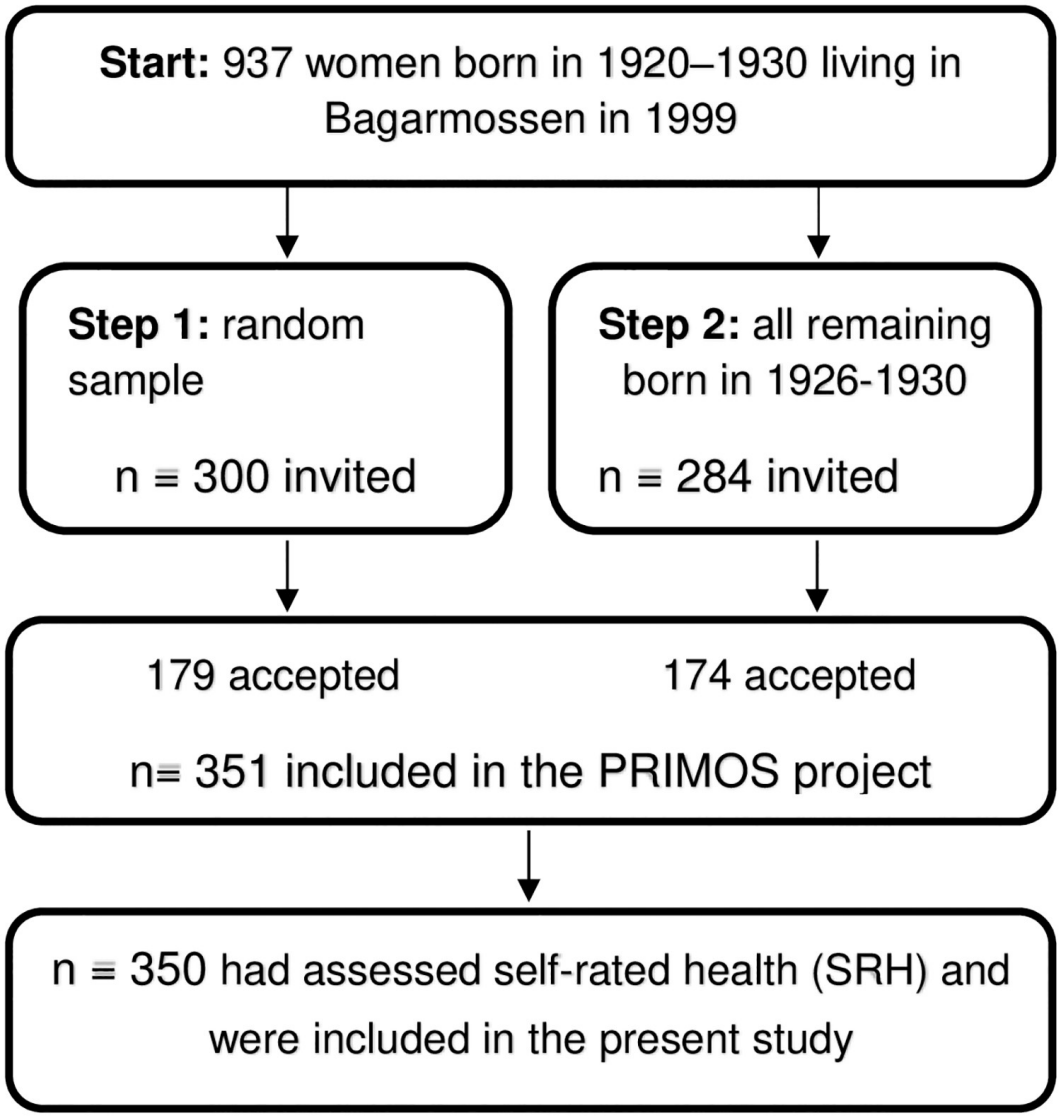
Flow-chart of the study.

In 1999, 937 women born between 1920 and 1930 were living in Bagarmossen (Southern Stockholm, Sweden) were eligible for the study (Fig 1). The selection was made in two steps. First, a random sample of 300 women were invited to participate, and 179 accepted. Second, an invitation was sent to all 284 of the remaining women born between 1926 and 1930, of which 172 accepted. This gave a total of 351 participants out of 584 invited (60%). There were data on SRH at baseline in 350 of them and they were included in the present study. There were no inclusion or exclusion criteria except that they had to be able to get to the primary health care centre for the study visit. See Fig 1. A non-participant questionnaire was sent to women who declined participation, and this was answered by 46.4%.

### Variables

All women were examined at baseline by the same physician.

*Age*: This was measured in years.

*Body mass index (BMI)*: This was calculated as kg/m^2^.

*One-leg standing time (OLST)*: This was measured in seconds for the best of four attempts, two on each leg, to stand on one leg for up to 30 seconds with eyes open, barefoot, and arms held alongside the body. This variable was dichotomized to whether or not they managed to stand for 10 seconds or not as earlier studies indicated that this is a relevant cut-off regarding future risk of hip fracture [35, 40, 41].

*Gait speed*: The time it took to walk back and forth (15 m × 2, including a 180-degree turn) as quickly as possible with shoes on in a well-lit corridor on an even floor was measured [37].

*Ability to get up from a chair without support*: Dichotomous variable where participants were asked to stand up from a sitting position without supporting themselves on the armrests of the chair [9, 42].

*Unable to rise from chair and slow gait speed*: Dichotomous variable combining gait speed less than 0.8 mps and inability to get up from a chair without support [13].

*Self-rated health (SRH)*: At baseline the participants answered the question “How would you rate your health right now” by putting a mark somewhere along a visual -analogue scale (VAS) ranging from “worst imaginable” to “best imaginable”. The distance between “worst imaginable” to the mark was measured in millimetres (min: 0 mm, max: 100 mm). This variable was divided into tertiles: low SRH, intermediate SRH, and high SRH.

Data were also collected via self-reported questionnaires or extracted from medical records on:

*Smoking status*: yes or no.

*Actual medication at baseline*: more than three drugs or not.

*Bisphosphonate therapy at baseline*: yes or no.

*Calcium and vitamin D supplement at baseline*: yes or no.

*Comorbidity*: more than two diagnosis or not.

*Self-reported fractures*: previous fractures in life before inclusion, at all and after the age of 50.

*Bone mineral densitometry (BMD)*: Measurements were conducted using Hologic QDR 4500 DXA equipment (Hologic, Marlborough, MA, USA) between 1999 and 2001. The measurements were expressed as T-scores calculated according to the NHANES-III reference population [43].

The variables used in this study were the T-score of the femoral neck, dichotomized to ≤−2.5 or not (indicating osteoporosis or not), and the total BMD of the femoral neck in g/cm^2^ as a continuous variable.

*Fracture risk assessment tool (FRAX*^®^*)*: A widely used algorithm giving the 10-year probability of fragility fractures [44–47]. The calculations were done in 2013.

### Statistical analysis

Only participants with data for SRH were included in this analysis (n = 350). In studies dealing with SRH, there are different strategies for how to present the data [19, 22]. Our variable SRH ranged between 0 and 100, but there was not necessarily equidistance between the values, eg an assessment of 50 is not necessarily twice as good as 25 in an individual. There were also no natural cut off levels.

Furthermore, SRH was skewed to the right. When looking at the variable in a histogram, the distribution visually seemed to be three-modal. We therefore chose to divide the participants into three groups: low, intermediate, and high according to their assessed SRH. Low SRH ranged between 5–51 mm (n = 113), intermediate SRH ranged between 52–73 (n = 118) and high SRH ranged between 74–99 mm (n = 119). High SRH was used as the reference. All variables were tested for normality (with Q-Q-plots), and if skewed they are presented as the median and as inter-quartile range (IQR). Otherwise they are presented as mean values and standard deviations (SD). The Kruskal–Wallis test was used for comparison between the three groups of SRH for skewed variables, and the Chi^2^ test was used if the variables were dichotomous. If the frequency in one of the groups was lower than five, we used Fisher´s exact test. For comparisons between the three groups of SRH in BMI and gait speed, we used one-way ANOVA and Bartlett´s test for variance testing, assuming that the sample was normally distributed and the variance were homogenous. The relationships between SRH and hip fractures and mortality were tested with the Cox proportional hazards regression model in order to take time to event into account. Since our material is numerically limited analyses with many covariates at the same time are likely to be senseless so we chose to examine simple associations and add one variable at a time. We defined possible confounders as variables that altered the age-adjusted HR ≥10%. Mortality as a competing risk was tested with Fine and Gray cumulative incidence function using the STATA package “stcrreg”. We tested that the proportional hazard assumption was not violated and we tested for multicollinearity using the STATA packages “collin” and “estat phtest”. P-values less than 0.05 were considered significant. All analyses were performed with STATA statistical software version 14.2 (StataCorp. LLC, College Station, TX, USA).

## Results

**Fig 2:**
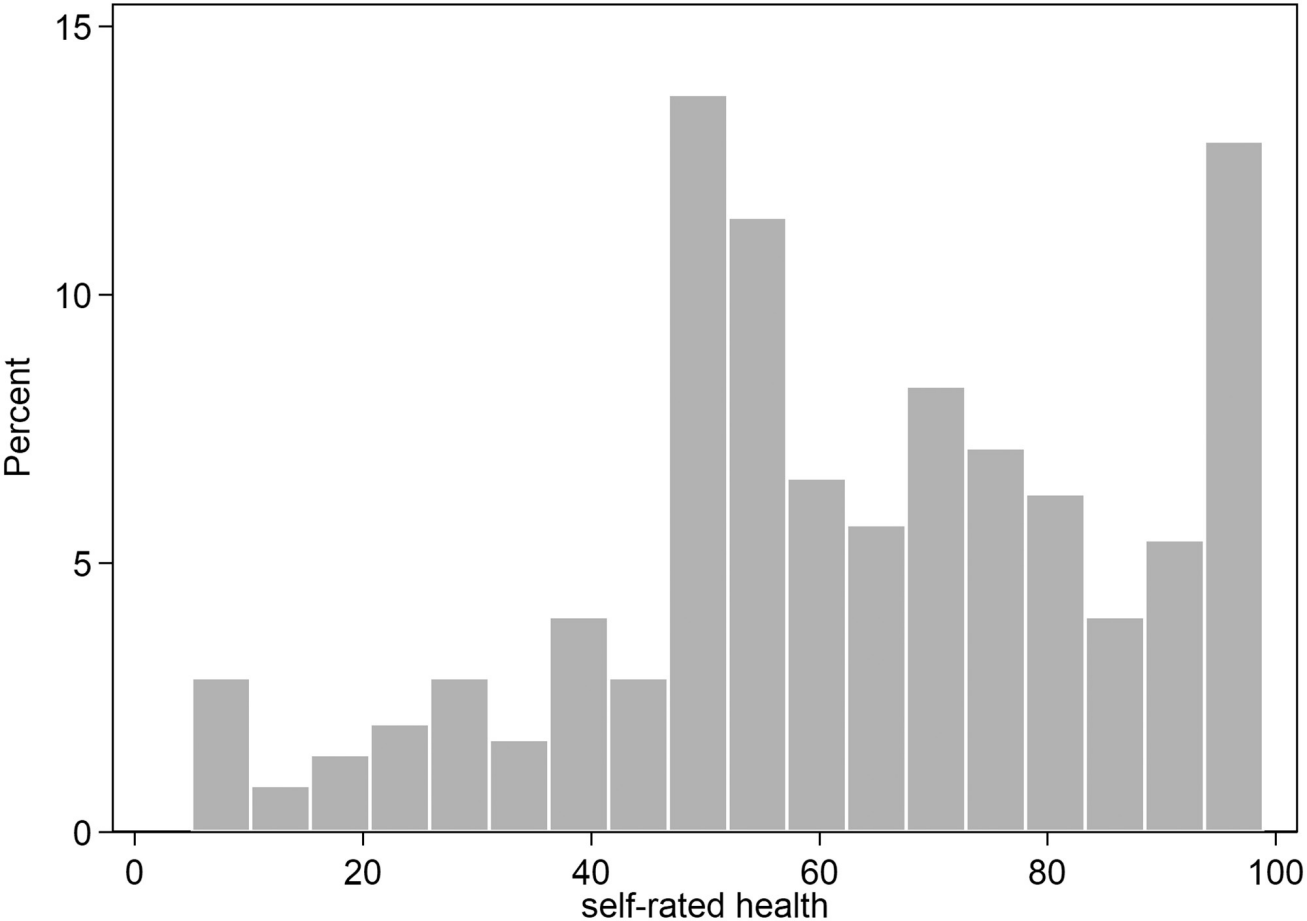
Percentage of distribution of SRH assessements (in mm). Cut-offs used for Self-rated health: Low 5–51 mm, Intermediate 52–73 mm and High 74–99 mm.

The women participating were all community-dwelling and aged between 69 and 79 years at baseline. The Non-participants questionnaire revealed that the participants were slightly younger (mean age 72.9 years vs. 73.9 years), reported previous hip fracture less often (1.4% vs. 4.6%p = 0.053), and reported previous wrist fracture more often (22% vs. 12%, p = 0.047). The proportion of subjects who considered their health to be as good as others of the same age was 35% in both participants and non-participants, but more women among the participants considered their health to be better than others of the same age (46.1% vs. 19.4%). Their total time at risk was 3,094.026 years with a median follow-up time of 9.8 years (IQR: 9.0–10.4 years). The median age at inclusion was 72.4 years (IQR 71.1–73.8). The median value of SRH was 62 mm (IQR 50–81 mm) (Fig 2).

The baseline characteristics are presented in Table 1.

**Table 1:** Baseline characteristics in the tertiles of SRH.

	Low SRH	Intermediate SRH	High SRH	p-value
**SRH range, (mm)**	5–51	52–73	74–99	
**n =**	113	118	119	
**Age, years, median (IQR)**	72.8 (71.4–74.3)	72.2 (71.0–73.8)	72.3 (71.1–73.7)	0.169^1^
**BMI, kg/m**^**2**^, **mean (SD)**	27.9 (4.6)	27.3 (4.0)	26.0 (4.8)	0.004^2^^,^ ^7^
**Gait speed m/s, mean (SD)**	1.3 (0.3)	1.4 (0.3)	1.5 (0.3)	< 0.001^2^^,^ ^7^
**OLST**^5^ **seconds, median (IQR)**	12 (5–30)	20 (8–30)	30 (12–30)	< 0.001^1^
**Smoking, yes n (%)**	16 (14.3%)	21 (18.0%)	19 (16.4%)	0.753^3^
**Drugs >3, yes n (%)**	44 (39.0%)	30 (25.4%)	11 (9.2%)	<0.001^3^
**Bisphosphonate therapy, yes, n (%)**	3 (2.7%)	3 (2.5%)	2 (1.7%)	0.824^4^
**Calcium and vitamin D supplement, yes n (%)**	13 (11.5%)	7 (5.9%)	6 (5.0%)	0.129^3^
**T-score at femoral neck (NHIII) ≤ −2.5, yes n (%)**	23 (21.1%)	26 (22.6%)	27 (23.5%)	0.911^3^
**Inability to stand up without using armrest, yes n (%)**	23 (20.4%)	12 (10.2%)	2 (1.7%)	< 0.001^4^
**Time spent outdoors >30 min/day, yes n (%)**	77 (68.1%)	108 (91.5%)	111 (93.3%)	<0.001^3^
**>2 diseases, yes n (%)**	46 (40.7%)	52 (44.1%)	22 (18.5%)	< 0.001^3^
**Self-reported hip fracture before inclusion, n (%)**	0 (0%)	3 (2.54%)	2 (1.8%)	0.334^4^
**Self-reported fracture after age of 50, n (%)**	33 (29.5%)	35 (29.9%)	39 (33.6%)	0.756^3^
**BMD**^6^ **at femoral neck, mean g/cm**^**2**^ **(SD)**	0.65 (0.10)	0.65 (0.11)	0.65 (0.10)	0.901^2^
**FRAX, % risk hip fracture, median (IQR)**	8.7 (6.1–14)	9.3 (5.4–15)	10 (5.9–15)	0.72^1^

^1^ Kruskal–Wallis.

^2^ One-way ANOVA.

^3^ Chi^2^ test.

^4^ Fisher´s exact test.

^5^ One leg standing time.

^6^ Bone mineral density.

^7^ ANOVA suggest differences between the groups of SRH regarding gait speed and BMI but not between which groups the difference lies. Also see the adjustments for these variables in the Cox proportional hazard regression model of association between self-rated health and hip fractures (Table 3).

There were differences between the tertiles of SRH at baseline in BMI (decreased as SRH increased). The tertiles did not differ significantly in age, BMD, FRAX^®^ result including T-score or T-score alone. There were differences in co-morbidity and having more than two diseases and more than three drugs were more common in the lower tertiles. The women who had assessed their health higher performed better in terms of gait speed, OLST, and the ability to get up from a chair without using the armrests and more often spent more than 30 minutes per day outdoors. There were only a few participants who were treated with bisphosphonates (n = 8) or calcium plus vitamin D (n = 26) at baseline, and for these variables there were no statistically significant differences between the tertiles. There were also no differences between the tertiles regarding self-reported fractures over age of 50, T-score less than −2.5 at the femoral neck, or bone mineral density at the femoral neck. Smoking was equally common in all tertiles. A total of 40 hip fractures occurred during follow-up, and the numbers differed significantly between the tertiles of SRH. There was a clear gradient across the SRH tertiles with most of the fractures among those who rated their health the worst. Out of 40 persons with hip fractures, 15 died over the study period, and their distribution did not differ significantly between the tertiles of SRH, nor did the deaths not caused by hip fracture or mortality in total (Table 2).

**Table 2:** Hip fractures, deaths, and survivals during the study in the tertiles of self-reported health (SRH).

SRH:	Low	Intermediate	High	p-value
n =	113	118	119	
**SRH, range (mm)**	5–51	52–73	74–99	
**At risk, years**	959.4	1,033.4	1,101.1	
**Hip fracture, n (%)**	18 (15.9)	16 (13.6)	6 (5.0)	0.020^1^
**Hip fracture and died, n (%)**	8 (44.4)	6 (37.5)	1 (16.7)	0.557^2^
**Died, total n (%)**	27 (23.9)	26 (22.0)	19 (16.0)	0.292^1^
**Survived, no hip fracture, n (%)**	76 (67.3)	82 (69.5)	95 (79.8)	0.072^1^

^1^ Chi^2^test.

^2^ Fisher’s exact test.

**Fig 3:**
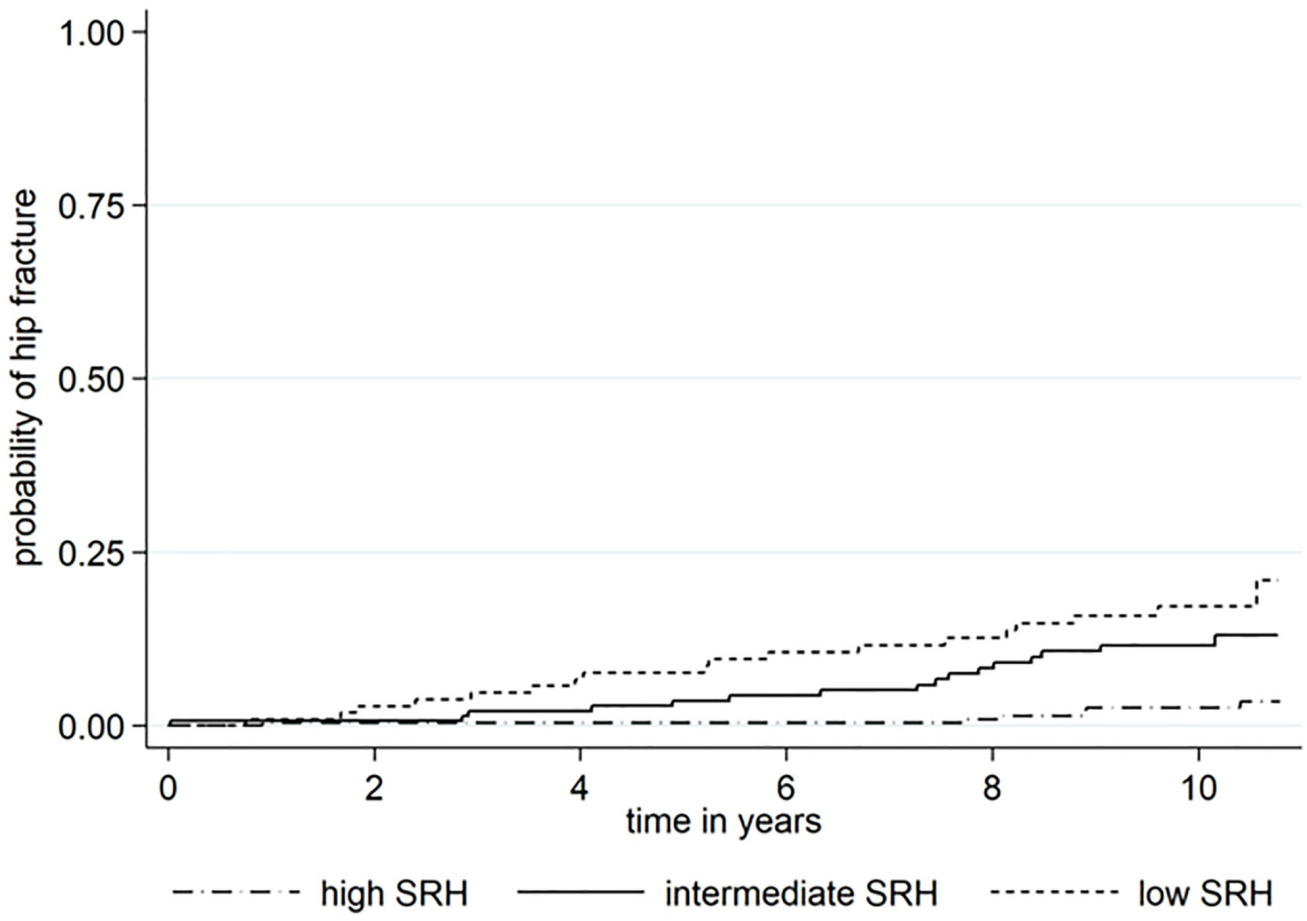
Kaplan Meier failure estimates for age 72.4 years (median age in the cohort).

The probability of hip fracture differed between the three groups of SRH as illustrated in a Kaplan–Meier failure estimate graph. The increase of probability of hip fracture in the group with low SRH also occurred earlier compared to those who assessed their SRH as intermediate or high (Fig 3).

The age-adjusted hazard ratio (HR) of suffering a hip fracture was 3.17 in the low tertile and 2.75 in the intermediate tertile compared with the high tertile.

The difference was even larger, HR 4.08 and 3.10 respectively, when we also adjusted for bone mineral density at the femoral neck as a continuous variable. Using a dichotomous variable with bone mineral density at the femoral neck (T-score) assessed as below −2.5 or not, HR was 4.04 and 3.18, respectively. Adjusting for smoking and BMI did not affect the significance of the original model (see Table 3). We obtained inconsistent results regarding the markers of risk of falling. When we adjusted the association between SRH and the risk of hip fracture with the ability to stand on one leg for more than 10 seconds, there was a significant threefold increase in the risk of hip fracture in the low and intermediate tertiles of SRH compared with the reference group (high SRH). When we instead adjusted the association with gait speed as a continuous variable there were no statistically significant difference comparing the tertiles of SRH. Adjusting for the ability to rise from a chair without using the armrest, there was only a statistically significant difference comparing the tertile with low SRH and the reference. Adjusting for a dichotomous variable combining inability to rise from a chair without using the armrests and gait speed less than 0.8 mps gave an age adjusted HR of suffering a hip fracture of 3.49 (p = 0.042) in the low tertile and 2.92 (p = 0.016) in the intermediate tertile compared with the high tertile (Table 3). We also analysed mortality as a competing risk to the age adjusted association between SRH and hip fracture. Subdistribution of hazards did not differ much from HRs so therefore we chose to use Cox proportional hazard regression model.

**Table 3:** Cox proportional hazards regression model testing the association between self-rated health (SRH) and hip fracture.

Outcome: Hip fracture	n =	High SRH	Intermediate SRH	Low SRH
Variables added:	-	**-**	-	**-**
**SRH**	350	(reference)	**2.86 (**95% CI 1.12–7.31)	**3.48 (**95% CI 1.38–8.77)
**SRH+ age**	350	(reference)	**2.75** (95% CI 1.08–7.04)	**3.17** (95% CI 1.25–8.01)
**SRH+age + >2 diseases**	350	(reference)	2.52 **(**95% CI 0.96–6.56)	**2.96** (95% CI 1.16–7.58)
**SRH+age + chair**^1^	350	(reference)	2.53 (95% CI 0.98–6.53)	**2.79** (95% CI 1.08–7.17)
**SRH+age + >3 drugs**	350	(reference)	**2.61** (95% CI 1.02–6.72)	**2.81** (95% CI 1.08–7.32)
**SRH+age + osteoporosis**^2^	339	(reference)	**3.18** (95% CI 1.15–8.77)	**4.04** (95% CI 1.48–11.02)
**SRH+age + Bone mineral density**^3^	339	(reference)	**3.10** (95% CI 1.13–8.55)	**4.08** (95% CI 1.50–11.16)
**SRH+age + outdoor**^4^	350	(reference)	**2.78** (95% CI 1.09–7.12)	**3.35** (95% CI 1.30–8.62)
**SRH+age + BMI**	350	(reference)	**2.84** (95% CI 1.10–7.33)	**3.32** (95% CI 1.29–8.50)
**SRH+age + smoking**	350	(reference)	**3.05** (95% CI 1.11–8.40)	**3.83** (95% CI 1.42–10.35)
**SRH+age + gait speed**^5^	346	(reference)	1.86 **(**95% CI (0.71–4.88)	1.67 (95% CI 0.62–4.50)
**SRH+age + OLST**^6^	348	(reference)	**2.60** (95% CI 1.02–6.65)	**2.80** (95% CI 1.10–7.13)
**SRH +age + marker of weakness**^7^	312	(reference)	**2.92** (95% CI 1.04–8.20)	**3.49** (95% CI 1.26–9.66)

^1^Inability to rise from a chair without using armrest.

^2^T-score <−2.5 at femoral neck.

^3^ at femoral neck.

^4^Spending more than 30 minutes outdoors every day.

^5^ mps.

^6^One leg standing time (OLST): Standing on one leg more than 10 s.

^7^ Unable to rise from chair without using armrest and gait speed less than 0.8 mps.

We found no association between SRH and mortality (Table 4).

**Table 4:** Cox proportional hazards regression model testing the association between self-rated health (SRH) and all-cause mortality.

Outcome: all-cause mortality	High SRH (reference)	Intermediate SRH	Low SRH
Variables added:			
**SRH**	(reference)	1.40 (95% CI 0.78–2.54)	1.57 (95% CI 0.87–2.82)
**SRH+ age**	(reference)	1.37 (95% CI 0.76–2.48)	1.50 (95% CI 0.83–2.70)
**SRH+ age+** ^1^		Not significant	Not significant

^1^ To the association between tertiles of SRH and all-cause mortality adjusted for age we also added one variable at time: having more than 2 diseases, inability to rise from a chair without using armrest, using more than 3 drugs, having T-score <−2.5, BMD, spending more than 30 minutes outdoors every day, BMI, smoking, gaitspeed (mps) and standing on one leg more than 10 seconds and marker of weakness but we found no significant association.

## Discussion

We found that the age-adjusted risk of suffering a hip fracture in our cohort of older and predominantly white women seemed to be three times higher if the participants assessed their health as low or intermediate compared to high at baseline (HR: 3.17 and 2.75, respectively). This was in line with our hypothesis that low assessed SRH could predict hip fracture in our cohort. In contrast to our second hypothesis, we found no association between SRH and mortality.

Earlier studies have also addressed SRH with different approaches and varying results. A prospective cohort with more than 9,000 white women over 65 years in the US explored risk factors for hip fracture and found an association between SRH at baseline and hip fracture during about four years of follow-up with HR: 1.7 (95% CI 1.3–2.2) towards poorer assessed health for each step between three SRH categories. The study subjects were very similar to ours [9]. In a later study from the same cohort, risk factors for hip fracture at the femoral neck and trochanteric region were compared and it was found that the two fracture types had partly different risk factors. Poor SRH predicted intertrochanteric fractures more strongly than femoral neck fractures during the follow-up of about eight years [48]. In our study we did not separate different kinds of hip fractures due to the small numbers of fractures in total (n = 40). A later published study on the same cohort above with a follow up of 10 years could not find any statistically significant association between SRH and hip fractures [49]. The authors considered that the differences between the results of earlier studies might be due to increased length of follow-up, an older cohort, loss of participants to follow-up, greater power to detect differences, or possibly random chance variations [49]. The results of our study contradict the first two explanations because we saw an association between SRH and hip fractures after 10 years in women that had a similar age at baseline. An association was also found between SRH at baseline and subsequent hip fracture in a six-year follow-up of older men and women of multi-ethnic origin with a crude rate ratio of 2.21 (95% CI = 1.03–4.72) [50]. The same subjects had previously been included in a larger study when they suffered their first hip fracture. In that study they found no association between (first) hip fracture and SRH. [51]. However, they considered the results from the two studies as congruent because another measure for impaired general health (being hospitalised the year before inclusion) stood out as a risk factor for primary fractures. Regarding the association between SRH and subsequent hip fracture, the authors considered collinearity between SRH and “reported problems with dizziness”. Both variables were significant in crude rate ratio analysis but showed no independent effect in an otherwise statistically significant multivariable model. These findings could be comparable with our inconsistent results after adding markers of risk of falling (OLST, gait speed, and ability to rise from a chair without using the armrest) to our age-adjusted model with SRH. We no longer saw a clear gradient between the SRH tertiles regarding hip fractures, and we did not get statistically significant results between all tertiles of SRH. We speculated that this might indicate that difficulties in balance are part of the decline in SRH and that this might partially explain the association with hip fracture that we observed, especially because it is well established that almost all fragility fractures of the hip are due to a fall from standing height or lower [10]. Gait speed and to rise from a chair are also part of the physical performance tests often used for diagnosis of sarcopenia [52]. Sarcopenia means a loss of muscle mass and muscle strength that leads to adverse events [13, 53]. When combining two of the markers for sarcopenia in one variable and adding that to the age adjusted model of the association between SRH and hip fractures the association remains. This is in line with the literature stating sarcopenia as an independent risk factor for hip fractures.

Several studies have also aimed at developing algorithms combining several risk factors for fracture prediction. In a prospective study with older women in the UK, they developed a risk score for predicting hip fracture risk for the next three years. Poor versus good SRH was associated with hip fracture with an OR of 4.1 (95% CI 1.14–14.72). SRH was a candidate for the final algorithm but was not included because the authors chose to replace it with variables they considered more specific [54]. A three-year prediction model for fragility fractures designed as a classification and regression tree analysis in postmenopausal women aged 50–64 in the US used fair or poor assessed health as one of three most important determinants in their final model with and without peripheral BMD [55]. Another five-year prediction tool for hip fracture was developed and valuated in a multi-ethnic longitudinal study of older women in the US. SRH was one of 11 items used in their final scoring model [56]. The three most recently mentioned studies developed models based on several risk factors, and because our study examined simple associations the results of those studies are not directly comparable to our study. However, they do highlight that SRH might be of interest in this context.

There are also studies where no significant independent association was found between SRH and hip fracture. Risk factors for hip fractures described by Cummings et al. (1995) were applied for hip fracture prediction in a prospective cohort of older women in Norway [9, 57]. The conclusion was that having more than five of the risk factors applied, including SRH, indicated a high five-year risk of hip fracture. Poor health itself, however, was not an independently significant predictor [9]. In a study of older men in the US there was no significant association between SRH and hip fracture [58].

The varying results regarding SRH and fragility fractures between different study populations might indicate that SRH is not a robust risk factor. Other studies have concluded that ratings of self-assessed health may vary between different demographic groups for example ethnicity, gender and age [59]. SRH is also by its nature a subjective measure. Also there are differences in the incidence of hip fractures between countries and within countries regarding local geography and season [60, 61]. Our results and several of the studies mentioned above, however, support that there might be an association between SRH and hip fractures at least in the context of older white women who are known to be a group with high risk. In the Kaplan Meier estimates of probability of hip fracture there were a divergence of the graph for low assessed SRH after approximately two year (Fig 3). A decline in QoL about two years before hip fracture have also been described by Diehr and Ahmed [31, 57].

There has been extensive research on prediction of hip fracture risk, and the state of knowledge today suggests that clinical risk factors together with BMD might be the best way to go.

When we adjusted for BMD in our study, this made the association between SRH and hip fracture stronger. This might indicate that SRH is not a proxy for BMD. Another study explored whether SRH could predict total hip low bone mineral density but could not find an association except in white non-obese men [59]. These findings might indicate that SRH adds information of fracture risk to the BMD measure at least among women. It is especially important since fragility fractures are known to occur in persons with non-osteoporotic BMD as well [45].

Initially, we were a bit concerned that BMD did not differ between SRH tertiles at baseline but affected the association between SRH and hip fracture in the final analysis. One possible explanation for this is that time to hip fracture is taken into account using Cox proportional hazards regression model. Fragility fractures may have occurred earlier in persons with low BMD compared to those with normal BMD. Another explanation might be that not all persons with low BMD suffered a hip fracture but most persons with hip fracture had low BMD.

We did not find an association between SRH and mortality nor any significant difference in mortality in the hip fracture patients between the tertiles of SRH (even though there seemed to be a trend). This finding was unexpected because association between SRH and mortality is widely accepted [25, 26]. Solbakken et al. also found that self-perceived health measured years before hip fracture predicted excess post-fracture mortality [62]. This might be due to that only 72 women died in total and 15 women with hip fracture died in our study, so the lack of a significant association could be due to lack of power.

### Strengths and limitations

The strengths with our study were that it had a longitudinal design, a long follow-up time (median 9.8 years), and that we had SRH assessments on all study subjects except one. Comprehensive registers in Sweden made it possible to retrieve outcome data on all our subjects so we had no loss to follow-up [38, 39]. Limitations of the study might be the limited size of the cohort and that the confidence intervals for hip fracture were wide. This leads to uncertainty about the size of the HRs even though they were statistically significant. The number of hip fractures (n = 40) during the follow-up corresponds well to the normative data in Sweden for women of that age group [1]. The tool that was used for assessing SRH had advantages and disadvantages. Positive aspects of using a single-item measure are that it is easy to assess and not so time consuming and labour intensive for the participants as, for example, questionnaires. Our SRH measure is a generic measure. There are also osteoporosis-specific measures, but in our cohort we had subjects both with and without osteoporosis. The women in our study assessed their SRH by putting a mark somewhere along a VAS scale (0 mm–100 mm), giving us a value of SRH between 0 and 100. We chose to split the variable into tertiles as described above in the section about statistics. The most commonly used version of SRH in the literature is a five-point scale ranging from excellent to poor or similar, and the five categories are usually pooled into two or three categories. Published research addressing comparison between different measures of SRH states that some kind of rescaling is needed [19, 63]. Another more general drawback is that reliability of self-ratings might be affected in older persons due to age-related cognitive deterioration. Another limitation may be the generalizability of the study. We studied the association between SRH and hip fracture in older and predominantly white women from a specific region in Stockholm. It may not apply to other populations. In our non-participant questionnaire, it was revealed that participants were slightly younger, reported less previous hip fractures and to a higher content considered their health to be better than others of the same age. This may indicate that our sample of older women was healthier than women in general of that age.

Finally we analysed BMD values and T-score of the femoral neck solely. We did not consider BMD of other locations or microstructure of the skeleton.

## Conclusions

Because hip fracture is such a devastating complication of osteoporosis, it is urgent to identify those persons at risk, preferably before they suffer a fracture. SRH appears to be an interesting variable to explore further in this context, particularly because it seems to add information on fractur risk independently of bone mineral density. In the future, it would be interesting to measure SRH more than once to observe the natural trajectory of SRH relative to the risk of hip fracture. This is also interesting because short-time fracture prediction might be more relevant in the oldest old. It is also worth keeping in mind that SRH might vary between ethnic groups, and this should be explored further.

## Supporting information

S1 Appendix (XLSX)
